# “It was one of those complicated cases”: health practitioners’ perspectives and practices of providing end-of-life care for people with profound intellectual and multiple disability

**DOI:** 10.1186/s12904-021-00873-5

**Published:** 2021-11-12

**Authors:** Hille Voss, April Loxton, Julie Anderson, Joanne Watson

**Affiliations:** 1 Netherlands Institute for Health Services Research (Nivel), Otterstraat 118, Utrecht, 3513 CR The Netherlands; 2 Deakin University’s School of Health and Social Development, Faculty of Health, 221 Burwood Hwy, Burwood, Melbourne, VIC 3125 Australia

**Keywords:** End-of-life, Profound intellectual and multiple disabilities, Severe and profound intellectual disabilities, Supported decision-making, Palliative care, Health practitioner perspectives, Complex support needs

## Abstract

**Background:**

Due to developments in health and social care, people with profound intellectual and multiple disability (PIMD) are living longer than ever before, meaning they are increasingly experiencing life-threatening health conditions requiring palliative care. Little is known about providing end-of-life care for people with PIMD. The aim of this study was to explore health practitioners’ perspectives and practices relating to end-of-life decision-making and planning for people with PIMD.

**Methods:**

Seven in-depth semi-structured interviews were conducted with health practitioners employed in a range of hospital and community services throughout Melbourne, Australia. Questions were designed to gather information about their experience, perceptions, and attitudes relating to people with PIMD during and at the end of their life. Each interview, ranging from 40 to 60 min in length, was audio recorded and transcribed. Inductive thematic analysis was used to analyse the data.

**Results:**

Four main themes emerged: limited participation, bias, dignity, and quality of death. Health practitioners indicated that people with PIMD are frequently excluded from participating in decision-making related to end-of-life care. Participants discussed reasons for this exclusion including challenges with communication and cognition. Participants reported a need for additional support and guidance in providing care for people with PIMD at the end of life. Professional and family bias played a role in end-of-life decision-making for people with PIMD. Participants reported a disproportional focus by palliative care practitioners on physical as opposed to emotional and spiritual well-being for patients with PIMD at the end of life. Finally, participants reported that people with PIMD generally did not die in specialised palliative care settings, but in segregated supported living environments.

**Conclusions:**

Due to negative perceptions of a person with PIMD’s decision-making capacity, people with PIMD are likely to be assessed as unable to express choice and preference regarding end-of-life care and are offered limited opportunity to be involved in their own end-of-life care. This research provides guidance for the development of training and professional development relating to people with PIMD at the end of life. It is hoped that this will increase the accessibility of end-of-life services for people with PIMD, ensuring that a respectful and dignified death can be a reality for all humankind regardless of disability.

**Supplementary Information:**

The online version contains supplementary material available at 10.1186/s12904-021-00873-5.

## Background

Due to developments in health and social care, people are living longer lives than ever before, including people with intellectual disability (ID). ID can be characterised by significant limitations both in intellectual functioning and in adaptive functioning [1, 2]. The levels of severity of ID and the need for support can vary from mild to severe/profound. People with Profound Intellectual and Multiple Disability (PIMD) are likely to have lifelong complex communication and physical support needs [3]. In addition to these complex support needs, people with PIMD are likely to have associated medical conditions (e.g., cerebral palsy, epilepsy, dysphagia, and respiratory disorders), which have historically resulted in them dying before their family carers [4].

An increase in life expectancy means that people with PIMD are now frequently outliving their family carers, resulting in a greater need for the support of palliative care services [5–7]. This creates a challenge for those providing support within the context of palliative care and end-of-life decision-making for people with PIMD, a group that have traditionally not had access to such services [8–10]. Due to limited experience supporting people with PIMD, health practitioners, including palliative care specialists, may lack the necessary skills to work effectively with this group [11]. Health practitioners, like a large proportion of society, may also have the view that without cognitive abilities such as intentional, rational behaviour and self-consciousness, characterised by some as defining personhood [12, 13], do not share the moral status of those with lesser degrees of cognitive disability. This results in a belief that autonomy, a concept that makes us uniquely human, has little relevance to people with PIMD.

As a consequence, medical decisions, including end-of-life decisions, are often made without consideration of the wishes of a person with PIMD in mind, via the use of substitute decision-making [14–16]. In addition, research on advance care planning (ACP), the process of defining, discussing, recording, and reviewing goals and preferences for future care [17], highlights that the end-of-life wishes and preferences of people with ID, especially of people with PIMD, are often not documented or discussed [18].

There is clear consensus in the empirical and practice literature of the value of decision-making autonomy at the end of life, in creating a “good death” for all of humankind [19, 20]. Despite this consensus, there is a widely held view that people with PIMD lack the ability to participate in decisions, including at the end of life [21]. “Historically, people with an intellectual disability have been assumed to be incapable of exercising the sort of control over their own lives which others take for granted” [22]. Jenkinson and Nelms make the point: “since by definition intellectual disability is characterised by significant impairments in adaptive behaviour, discretion, social competence, and comprehension of own self-interest, the temptation has been to presume total incompetence in decision-making” [23]. This negative perception is particularly apparent for people with PIMD. Such views are in conflict with the United Nations Convention on the Rights of Persons with Disabilities (UNCRPD), Article 12, that mandates signatory nations’ recognition of the universal right to autonomy on an equal basis with others in all aspects of life (including at the end of life) [24, 25].

Watson [26] found that a key factor underlying supporters’ willingness and ability to engage in supported decision-making with people with PIMD was their attitude toward the person they were supporting. Specifically, she found that where a supporter had a positive perception of the person they were supporting in terms of their personhood and their ability to live an autonomous life, the more responsive they were to the person’s expression of will and preference [26]. Building on this research, the authors of this paper explored the attitudes of health practitioners regarding end-of-life care for people with PIMD. To ensure a respectful and dignified death for all, it is important to gain insight into how health practitioners’ perspectives of people with PIMD play a role in end-of-life decision-making and planning.

## Methods

### Aim

The aim of this study was to explore health practitioners’ perspectives and practice concerning people with PIMD, particularly in relation to end-of-life decision-making and planning.

### Data collection

Seven in-depth semi-structured interviews were conducted over Skype between September and December 2019. An interview schedule, consisting of a variety of open-ended questions was developed and used to guide the interviewer (see Additional file 1). The questions were designed to elicit information about health practitioners experiences, perceptions, and attitudes relating to people with PIMD both during and at the end of their life. The interviews ranged from 40 to 60 min in length. Each interview was audio recorded and transcribed by an external transcription service. The participants were de-identified.

### Data analysis

**Fig. 1:**
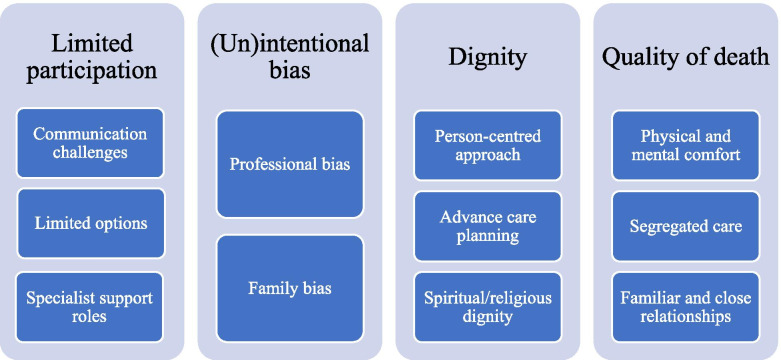
Themes and sub-themes

The authors collaboratively engaged in a process of inductive thematic analysis. This involved engaging in a six-step process as outlined by Braun and Clarke [27]. The authors initially familiarised themselves with the data, without relying on an existing coding frame [27]. Two researchers independently reviewed the qualitative data, making notes about what was commonly discussed or addressed throughout each of the interviews, while also determining how it related to the research aims and questions. During the initial readings of the transcripts, researchers further identified the discourse prevalence, agreeing that any comments or opinions that were repeated more than once within or across the interviews, were noted as an important code for later analysis and review. After initial notetaking, transcripts were revisited, and a basic framework of codes and overarching themes were created. At this stage, the transcripts were then entered into NVIVO, a qualitative data management software, and revised for a third time. Within NVIVO, the transcripts were then re-coded and checked against the initial coding framework established in the first two stages of analysis, and any changes to the coding were confirmed within NVIVO. Utilising knowledge of the extant literature and the research field, codes were then assigned to an overarching theme, taking into consideration findings from prior literature. It was at this point that the overarching themes were also reviewed and re-named to reflect the data and prior literature more accurately [27]. A total of four overarching themes were agreed upon, with 11 relevant sub-themes (see Fig. 1).

### Participants/recruitment

Participants were recruited via an advertisement published in an online newsletter distributed by Palliative Care Victoria to its members, many of whom are palliative care professionals. Seven practitioners responded to the advertisement. These practitioners were in a range of hospital and community services throughout Melbourne, Australia. All participants identified as Australian, one identifying as Caucasian, and two describing their ethnic background as Vietnamese and Cypriot Greek. Ages ranged from 36 to 52 years, with an average age of 45.6 (one participant did not disclose their age). Practitioner professions included two social workers, two nurses, a pharmacist, an Occupational Therapist (OT) and a physiotherapist. Inclusion criteria for participant recruitment included being proficient in English, and having experience as a health practitioner, either in palliative care, and/or supporting people with PIMD. The professional backgrounds of each participant are listed in Table 1.

**Table 1:** Professional background characteristics of participants

	Experience as health professional (years)	Experience in working with people with intellectual disability (years)
*Participant_01*	≥20	≥20
*Participant_02*	≥30	<5
*Participant_03*	≥30	≥30
*Participant_04*	≥10	<10
*Participant_05*	≥10	≥10
*Participant_06*	≥25	≥25
*Participant_07*	≥15	<5

## Results

Qualitative results highlighted a diverse range of health practitioners’ experiences and views on the topic of end-of-life planning for people with PIMD. Salient perspectives among health practitioners centred on four main themes, and a range of sub-themes (see Fig. 1). These included limited participation, (un)intentional biases, dignity, and quality of death.

### Limited participation

People with PIMD were reported to be routinely excluded from participating in decision-making related to their end-of-life planning and care. Health care practitioners discussed various reasons for this exclusion, all centring around people with PIMD being perceived as ‘different’ to the rest of the population. “*A person with cognitive impairment around end-of-life decision-making, it’s quite different to when people actually have full capacity to make decisions”* (Practitioner_01).

#### Communication challenges

According to health practitioners, it is difficult to assess the ability of people with PIMD to express choice and preference regarding end-of-life planning and care. Interviewees identified some of these challenges. Firstly, they identified a lack of attention to communication within the assessment process.

Practitioners highlighted that a person “*may have an effective way [of communicating], but it might not be effective for the assessor*” (Practitioner_04). Practitioners highlighted the importance of not assuming people with PIMD have no decision-making capacity because they have complex communication needs. “*You know, people might have a pod or some sort of augmentative alternative communication device. So don’t make assumptions that just because they don’t speak…that they don’t communicate”* (Practitioner_03).

Interviewees identified the need to use alternative forms of assessment for determining the decision-making capacity of people with PIMD. Such forms included assessing “*ability to respond appropriately, be orientated to time and place, know where they are”* (Practitioner_02)*,* use *“eyes and grimacing and other facial movements”* (Practitioner_03) in consistent responses to practitioner questions*,* or having “*some understanding of the consequences of your decision”* (Practitioner_05).

Judgements on decision-making capacity were further noted as inappropriate in the context of PIMD as tests focused primarily on decision-making capacity through behaviours that may not be achievable for some, such as handwriting:

*I think it's difficult to determine somebody's capacity to make decisions and it's contextual and I think it's on the back of really poor standardised [practises] as well. Which aren't geared for people with intellectual disabilities, not necessarily. A lot of the neuropsychology battery of tests they use involve handwriting... I’d confidently say that I don’t know any service... I’m not saying it doesn’t exist...that is able to determine capacity in just whether or not a person has true capacity decision-making in the disability sector. And people are very weary of going into that space because it’s complicated* (Practitioner_04).

#### Limited options

Participants reported that decisions about the lives of people with PIMD were generally made by a family member or health practitioner, without the input of the person themselves. According to interviewed health practitioners, this is based on the belief of others that people who are unable to communicate through traditional means such as speech, should be absent from decisions impacting their lives:

*I guess that goes back to that initial statement I made about the shift in dignity. When we think about what we want around us and we’re given quite good choice. We’re able to explore the choice of, oh, do we want to be at home to die? Do we want to be in the hospital to die? Do we want to go to a special unit or all those sorts of things. Often those are the decisions we get to make quite happily. Whereas people with intellectual disabilities don’t get to make those decisions… So we may stay at home even longer or they may get put into a facility or into a palliative care unit to just wait* (Practitioner_01).

Practitioners also noted that there was “*a narrower set of options*” available to people with PIMD (Practitioner_04), based on assumptions that a variety of options would not be relevant or desired by them. For example assumptions such as *“You probably don’t want that because why would that matter to you? You’re a person with a disability, you’re probably not going to gain much from it, so, therefore, I’m not going to give it to you.”* (Practitioner_04).

#### Specialised support roles

In light of the challenges surrounding end-of-life planning and decision-making, practitioners reported a desire to receive additional support, with some suggesting the development of a specialised service role to help families, professionals and individuals come to decisions based on the wishes of the individual with PIMD:

*Ultimately what I would like to see happen is…people that specialise in intellectual disability and end-of-life care. And that when a diagnosis of that comes up, that there are, almost advocates and supporters of people with intellectual disability to explore every option from a perspective of rights-based on what they’re entitled to… so that people with intellectual disabilities aren’t left out of the bigger picture* (Practitioner_01).

The inclusion of such a role may also assist in supporting health practitioners, by offering external advice and guidance, and acting as a liaison between the health practitioners, the family and the individual with PIMD:

*It’s great to have family and professionals there. But sometimes I think they all come in with their own biases, and that’s where I think sometimes an extra consultant or a person that can actually advocate on behalf of the person, who has the person at centre to assist with some of that planning, decision-making [and] information sharing* (Practitioner_01).

The desire for a specialised role or additional support in end-of-life decision-making and planning stems from practitioners’ expressing concern over the skills they lacked in supporting people with PIMD at the end of life, where additional training or support may assist in future preparation for end-of-life care and inclusive practice:

*... I don’t think I really have very good skills in that area to be honest. I think it would be great to have some facilitators to come in and assist. It’s such a specialised area* (Practitioner_02).

### (Un)intentional bias

Participants discussed the existence of personal and professional unintentional and intentional bias within the context of end-of-life care for people with PIMD.

#### Professional bias

Observations of professional bias were illustrated through choices being “*talked to them, I say talk to them. Not necessarily with them*” (Practitioner_04). Moreover, health practitioners and doctors were viewed as inappropriate decision-makers for people coming to the end of life as their interactions and knowledge on the individual were limited and time restricted:

*I truly believe that. I feel like external professionals shouldn’t be involved in, I feel like perhaps I would say in the medical model, often the information that is given to them is made on assumptions* (Practitioner_04).

The snap-decisions made by professionals were also found to be negligent towards individuals with disability or the families’ preferred choices, resulting in health practitioner only seeing “*this really sick person…they don’t see what they’re like when they’re well, they’re making a big judgement call*” (Practitioner_05). According to the interviewees, some professionals also felt conversations about wishes and preferences for the end of life were too difficult or time consuming to have:

*I think none of the doctors wanted to sit down and have the discussion and make the decision with the family because a lot of doctors find it quite confronting and hard, at the best of times… [The doctor] was just asked to do this procedure and he didn’t question it because he just didn’t want to get involved I guess… [The doctor] just had a task to do and he wanted to get it done and move onto the next task, but I think that’s what the difference sometimes is between doctors and nurses, we look at ourselves as more of an advocate for the patient at times when the doctors have their blinkers on* (Practitioner_06).

#### Family bias

In addition to professional bias, personal and familial biases were also a challenge. Practitioners noted significant power imbalances between people with PIMD and their family members, as even when the individual was capable of making decisions, families would undermine their choices, preferring outcomes that were beneficial to them, not the individual:

*We find often it’s the family or the parent that essentially makes the decision even if the person themselves is able to communicate that they don’t want that, we do often experience a parent overriding that wish or verbalisation, you know, or communication and just decide on their behalf* (Practitioner_04).

Dominant family decision-making in these situations could be attributed to the habit of caring for a loved one all their life and are used to making these decisions for them on a regular basis. Health practitioners experienced that family members inadvertently prioritise their decisions on assumptions that the person is incapable, or not understanding of the situation:

*The person who is actually, going through their own end of life, they’re the main person who should be involved. And it should really be around supporting that person to be able to make a decision no matter what capacity they have. And it should always be like that… I mean, the challenge with that is often people, particularly with an intellectual disability, are perceived as children, even though they’re an adult…I think that’s when the parents tend to, or the family tends to, come in and make an executive decision because they still see themselves as very much a guardian.* (Practitioner_04).

As interviewees noted, the end-of-life process can be traumatic and stressful, particularly when the family may not be ready to say goodbye. Interview data highlighted that “*loved ones don’t always make the most rational decisions*” (Practitioner_03) and family members found it difficult to engage in discussions about end-of-life care:

*With not much success though because all we can do is just suggest and advise and sometimes it takes a long time…it will take years of the doctor and the team suggesting to the family that perhaps a different way of being, or a different way of living for the person, you know, would make the person more comfortable* (Practitioner_07).

### Dignity

Dignity, as discussed by practitioners, centred on allowing the person with PIMD to control their life and plan their desired death. The following section covers the importance of maintaining dignity in end-of-life care through the advocacy of person-centred approaches and the importance of fulfilling spiritual and religious preferences.

#### Person-centred approach

People with PIMD should be central to the planning process towards their end of life, as “*dignity is about inclusion in the process and really trying to understand where the person might be*” (Practitioner_01). Practitioners did, however, note that end-of-life wishes and preferences of people with PIMD and “*decisions of funerals, donations of body and all those other thoughts that need to come up to that*” (Practitioner_01) are not easy to determine. Nevertheless, health practitioners emphasised that these challenges should not be an excuse for families and professionals to override end-of-life decisions, instead those involved should at least attempt to communicate with the individual, recognise their way of communication and interpret their wishes and needs:

*So no matter what, any attempt, it doesn’t really matter. The attempt is what matters. You might not get something that is what people would deem a quality response, but that’s not the point. The point is to actually try and be able to ascertain and not just make the assumption that they can’t participate in any aspects of the end of life* (Practitioner_04).

#### Advance care planning

Additional barriers facing health practitioners and the confirmation of end-of-life planning included the issue of deterioration through age, illness, and time. Participants recommended early discussions with patients “*before they deteriorate*” (Practitioner_02), establishing plans and goals before communication dissipates:

*What we would like for our area is really early referrals, to get to know the person before they start to deteriorate. So when they’re well and they’ve got a bit more capacity, or whether, when they can engage, I mean I think that’s a huge area that we’ve always, and we’ve identified and we know* (Practitioner_02).

Early planning can also present better outcomes closer to the end of life, as decisions have already been made, allowing the person and their family to be in the moment rather than coping with the emotional strain of planning everything in a timed or rushed environment:

*Devise a plan so that when it comes to the person’s health deteriorating to the point where they are no longer able to have a decent quality of life, they don’t have to be making a huge decision on the spot during the time of crisis and during a time of extreme grief* (Practitioner_07).

#### Spiritual/ religious dignity

Though limited in its discussion, religious and spiritual dignity was also presented as an important element in end-of-life care. As results have indicated, people with PIMD are often declined the basic opportunities as “*we forget that there’s other dimensions to their lives and…those social, emotional and spiritual aspects of our existence*” (Practitioner_02). The inclusion and provision of spiritual or religious support could further enhance the quality of end-of-life care for people with PIMD:

*Pastoral care is a service that is available to people in hospitals. It tends to be very much part of the palliative care units, but also in end of life they are meant to be an external party that is very much around spirituality…I feel like we don’t enable that type of service to occur in our services, but certainly I don’t feel like the pastoral care service understands how to support people with disabilities (Practitioner_04).*

### Quality of death

Final discussions noted by health practitioners placed the utmost importance on providing care based on the quality of death through physical and mental comfort, and familiar and close relationships for people with PIMD. Quality of death does not only focus on the process of death but includes end-of-life planning and assessing, caring for, supporting, and assisting persons who are terminally ill.

#### Physical and mental comfort

Thematic data on quality of death was commonly associated with physical comfort for people nearing the end of life, through the avoidance of pain, emotional distress and maintaining comfort. One interviewee noted that “*I think keeping the person comfortable, physically comfortable, is probably what should be adhered to the most*” (Practitioner_07). Practitioners reiterated aims of maintaining an individual’s comfort, even when difficult decisions concerning medical procedures or hospital admission were made to prioritise a persons’ quality of life:

*It was all about quality of life really. When it was clear that her quality of life wasn’t getting any better and that, you know, a hospital admission would be distressing and disruptive and it would probably make her go backwards and increase her risk of infection* (Practitioner_03).

The significance of physical and mental comfort was also deemed equally important with health practitioners acknowledging that hospitals can be “*such different environments for anyone, let alone someone with a profound disability*” (Practitioner_02),

#### Segregated care

In preparation for the end of life, health practitioners discussed the additional impacts of segregated care for people with PIMD, where individuals were removed from their share homes, away from their friends and familiar carers, and into a new and strange environment that may induce more stress and anxiety:

*We would take them out of their own environments. They don’t get to see their friends and the people that they know and even their family contact is sometimes considerably reduced by their location… they actually moved [patients] out of the facilities that I was in, to go into aged care facilities because of the concern around staff. But that just meant that the staff, the people that visited, the other residents, none of them got to see that person in the end and that person was left quite isolated* (Practitioner_01).

In addition to removing a person from their home, their friends and networks are also impacted by the separation, where house mates became depressed when they were unable to visit, say goodbye or grieve – all of which are important steps when processing death and receiving closure:

*One of my concerns is about how we hide people with intellectual disability from death…I’ve worked in facilities where people have died before and the residents, they haven’t even been able to go to the funeral. There’s been very limited conversation about death and dying … And I think that actually robs them of their dignity around grief and loss… to be able to have that conversation with the people around them is a very valuable thing* (Practitioner_01).

#### Familiar and close relationships

Providing end-of-life care and ensuring a quality death was also said to be influenced by quality relationships, reiterating the importance of familiarity and comfort through connections:

*If you are surrounded by people who know you well and you’re in a familiar environment, you will do a lot better. It’s much less distressing* (Practitioner_03).

Knowing the individual well and building close relationships is also important to accurately interpret communication and act upon wishes and needs. Such relationships did not always require family associations, as close connections with carers were just as vital and impactful:

*The carers are a fantastic resource, particularly if they build up a relationship over the years. Even when [person with PIMD] was in the facility, there was one carer who was involved in her care from, you know, the day she went in to the day she passed, and she knew [person with PIMD] inside out, which…was a great comfort…Then, you know, going back after she’d passed away, it was pretty clear that they missed her…and the staff as well* (Practitioner_03).

## Discussion

Health practitioners shared perspectives and practices highlighting the multiple challenges faced by people with PIMD to achieving an end-of-life experience reflective of their will and preference. Because of complex communication and support needs, people with PIMD are likely to be assessed as unable to express choice and preference regarding end-of-life care, and consequently are offered limited opportunity to be involved in end-of-life decision-making and planning. This phenomenon not only relates to participation in decision-making about end-of-life planning for people with PIMD but is reflected across the lifespan. Where professionals hold negative attitudes regarding a person with PIMD’s capacity to communicate their will and preference within the context of decision-making, their opportunities for participation and social inclusion in activities of their choice are reduced [28–31].

Person-centred end-of-life care for people with PIMD requires supporters’ willingness, time and effort of to get to know the person and to be able to recognise and interpret a person’s expression of will and preference [10, 21]. Practitioners included in this study stressed the importance of involving the person with PIMD in a similar way as people without disability, stating that “*every effort should be made to be inclusive*” (Practitioner_01). On the other hand, participants also admitted that supporting people with PIMD during or at the end of their life is *different* and *a specialised area*, as one of the interviewees noted: *“It was … one of those complicated cases”* (Practitioner_02). Health practitioners expressed a lack of confidence relating to their own skills and suggested facilitators, patient advocates or other specialised service roles to assist in end-of-life decision-making and planning for people with PIMD.

Health practitioners described their interactions and knowledge of working with people with PIMD as limited, and consequently viewed themselves as ill-equipped to support people with PIMD at the end of life. Additionally, in line with previous research, there was a tendency among professionals to avoid discussing end-of-life issues with people with ID [11, 32–34]. It is known that even when doctors know their patients with ID intimately, they are reluctant to discuss their own opinion of their patients’ quality of life and draw heavily on the opinion of family in end-of-life decision-making [35, 36]. To avoid bias and to accurately reflect a persons’ end-of-life wishes, it is important to involve everyone who knows the person with PIMD in decision-making and collaboratively interpret and act upon will and preferences [10, 18, 37].

Although familiar environments and quality relationships are of utmost importance for people with PIMD [10, 21, 38], health practitioners in this study reported that people with PIMD regularly die in segregated care settings, away from their share homes, friends, and familiar carers. In addition, as illustrated in previous research, health practitioners reported that people with PIMD are denied access to essential services in end-of-life care, including specialist palliative care and pastoral services [7, 39, 40]. Furthermore, the health practitioners in this study indicated that providing end-of-life care for people with PIMD focused on physical needs as opposed to social, emotional, or spiritual care needs, which were often not assessed or discussed for people with PIMD. Pastoral care extending beyond institutionalised traditions or religious backgrounds, in some countries such as the United Kingdom and the Netherlands, focusses on presence, empowerment, and bringing peace [41]. Increasing attention for and accessibility of these services focusing on psychosocial needs could greatly enhance the quality of end-of-life care for people with PIMD [42, 43].

The current research is limited to a relatively small number of health practitioners recruited from a range of hospital and community services throughout Melbourne, Australia. This may limit the generalisability of the study and comparisons to other regions outside the urban Australian context would need to be carefully considered. In addition, although attempts were made to recruit medical doctors for the study, none responded to the advertisement. Therefore, they were not recruited and consequently the views of medical doctors were not included in our study. A last limitation of this study concerns the fact that health practitioners voluntarily participated in the interviews. This means participants were interested in the study focusing on end-of-life care for people with PIMD and believed in its importance, and might have been more likely to respond in socially desirable ways.

In spite of these limitations, the authors are of the view that the obtained data provides valuable insight into health practitioners’ perspectives and practices with regard to end-of-life decision-making and planning for people with PIMD. This is a group of people who are often not able to express their views in conventional ways and who are overlooked and ignored in research and practice concerning end-of-life care [10, 21, 44]. Further research should encompass the needs and experiences of people with PIMD in end-of-life care and should be aimed at better understanding and overcoming health practitioners’ negative attitudes regarding the decision-making capacity of people with PIMD. This is especially relevant in the current context of the COVID-19 pandemic to ensure equitable access and treatment for people with PIMD. Moreover, future research could focus on exploring factors that support partnership between specialist palliative care providers and ID services, as well as possibilities for specialised service roles to assist in providing end-of-life care [45].

## Conclusions

This study provides valuable insights into a neglected field of research relating to end-of-life decision-making and planning for people with PIMD. The results show that, due to negative perceptions of a person with PIMD’s decision-making capacity, people with PIMD are likely to be assessed as unable to express choice and preference regarding end-of-life care and are offered limited opportunity to be involved in their own end-of-life care. Health practitioners have an important role in addressing end-of-life care needs and preferences but feel uncertain about their competencies in providing care for people with PIMD during or at the end of their life. There is a clear need for additional and specialised support in end-of-life decision-making and planning for people with PIMD. The provision of such support is common practice in some parts of the world. For example, in the Netherlands specialised palliative and pastoral care for people with ID is acknowledged and respected as a medical specialty. Positive attitudes toward the capacity of people with PIMD to participate in decisions about their end-of-life care, coupled with an increase in the accessibility of this care in the form of specialist support, such as palliative care professionals and pastoral care teams, are important for achieving equality for people with PIMD at the end of life.

## Supplementary Information

**Additional file 1.** Interview guide.

## Data Availability

Identifiable electronic data and the codes for data re-identification are stored on a password protected file on the Deakin University server and are not available to the public to protect participants privacy. De-identified transcripts of interviews are kept by the authors for 5 years and restrictions apply to the availability of this de-identified data, which were used by the authors specifically for this project with consent from study participants. The data analysed during the current study are not publicly available due to privacy and ethical concerns but are available from the corresponding author on reasonable request.

## Abbreviations

PIMD: Profound Intellectual and Multiple Disability
ID: Intellectual disability
