# Supplementary material for: “It was one of those complicated cases”: health practitioners’ perspectives and practices of providing end-of-life care for people with profound intellectual and multiple disability
Source: BMC Palliat Care. 2021 Nov 12;20:177. doi: 10.1186/s12904-021-00873-5 (PMC8586595; doi:10.1186/s12904-021-00873-5)
Supplement: Supplementary file 1 — Additional file 1. Interview guide. [file 12904_2021_873_MOESM1_ESM.docx]

## Additional file 1. Interview guide

1. *How long have you been working as a health professional, and in what capacity?*

1. *Have you had experience supporting someone with end-of-life decision making in your role as a health practitioner?*

| ***If so:*** | ***If not:*** |
| --- | --- |
| Describe these experiences | Describe what factors you think should be taken into consideration in decision-making and planning at the end of life. |
| Describe the context of the decision-making  *Who was involved? How were they involved? Do you think anyone should have been involved in another way?* | Describe who you think should be involved in this decision-making and planning. In what way should they be involved? |
| What factors have you found to be important in end-of-life decision making? | In your opinion, who should make the decision? |
| In your experience, who do you think should be involved in this decision-making and planning? In what way? | What does decision making capacity look like to you? |
| Who makes the decisions? / Who is responsible for the decisions? | Can everyone make their own decisions? Who can and who can’t? |

1. *Have you had experience supporting someone with severe intellectual disability with end-of-life decision-making and planning?*

| ***If so:*** | ***If not:*** |
| --- | --- |
| Describe these experiences  *How did the person communicate? Were their preferences heard and if so, how?* | Describe what factors you think should be taken into consideration in decision-making and planning for people with severe intellectual disability at the end of life. |
| Describe the context of the decision-making    *Who was involved? How were they involved? Do you think anyone should have been involved in another way?* | Describe who you think should be involved in this decision-making and planning. In what way should they be involved? |
| What factors play a role in this decision-making and planning? | Who should make the decisions? |
| In your experience, who do you think is best involved in this decision-making and planning? In what way? |  |
| Who do you think should make the decisions? |  |

1. *Can you describe a person that you would say can be involved in end-of-life* *decision-making and planning?*

- Describe the person

1. *Can you describe a person that you would say cannot be involved in end-of-life* *decision-making and planning?*

- Describe the person
